# Comparing Outcomes of Post-Cardiotomy Cardiogenic Shock Patients: On-Site Cannulation vs. Retrieval for V-A ECMO Support

**DOI:** 10.3390/jcm13113265

**Published:** 2024-05-31

**Authors:** Mircea R. Mihu, Ahmed M. El Banayosy, Michael D. Harper, Kaitlyn Cain, Marc O. Maybauer, Laura V. Swant, Joseph M. Brewer, Robert S. Schoaps, Ammar Sharif, Clayne Benson, Daniel R. Freno, Marshall T. Bell, John Chaffin, Charles C. Elkins, David W. Vanhooser, Aly El Banayosy

**Affiliations:** 1 Specialty Critical Care, Advanced Cardiac Care and Acute Circulatory Support, Nazih Zuhdi Transplant Institute, Integris Baptist Medical Center, Oklahoma City, OK 73112, USArobert.schoaps@integrishealth.org (R.S.S.);; 2 Department of Medicine, Oklahoma State University Health Science Center, Tulsa, OK 74077, USA; 3 Department of Surgical Critical Care, MedStar Washington Hospital Center, Washington, DC 20010, USA; 4 Department of Anesthesiology, Division of Critical Care Medicine, College of Medicine, University of Florida, Gainesville, FL 32610, USA; 5 Department of Anesthesiology and Intensive Care Medicine, Philipps University, 35043 Marburg, Germany; 6 Critical Care Research Group, The Prince Charles Hospital, The University of Queensland, Brisbane, QLD 4072, Australia; 7 Department of Cardio-Thoracic Surgery, Integris Heart Hospital, Integris Baptist Medical Center, Oklahoma City, OK 73112, USA

**Keywords:** post-cardiotomy shock, ECMO retrieval, V-A ECMO, ECLS, PCCS

## Abstract

**Background:** Post-cardiotomy cardiogenic shock (PCCS) remains a life-threatening complication after cardiac surgery. Extracorporeal membrane oxygenation (ECMO) represents the mainstay of mechanical circulatory support for PCCS; however, its availability is limited to larger experienced centers, leading to a mismatch between centers performing cardiac surgery and hospitals offering ECMO management beyond cannulation. We sought to evaluate the outcomes and complications of PCCS patients requiring veno-arterial (V-A) ECMO cannulated at our hospital compared to those cannulated at referral hospitals. **Methods:** A retrospective analysis of PCCS patients requiring V-A ECMO was conducted between October 2014 to December 2022. **Results:** A total of 121 PCCS patients required V-A ECMO support, of which 62 (51%) patients were cannulated at the referring institutions and retrieved (retrieved group), and 59 (49%) were cannulated at our hospital (on-site group). The baseline demographics and pre-ECMO variables were similar between groups, except retrieved patients had higher lactic acid levels (retrieved group: 8.5 mmol/L ± 5.8 vs. on-site group: 6.6 ± 5; *p* = 0.04). Coronary artery bypass graft was the most common surgical intervention (51% in the retrieved group vs. 47% in the on-site group). There was no difference in survival-to-discharge rates between the groups (45% in the retrieved group vs. 51% in the on-site group; *p* = 0.53) or in the rate of patient-related complications. **Conclusions:** PCCS patients retrieved on V-A ECMO can achieve similar outcomes as those cannulated at experienced centers. An established network in a hub-and-spoke model is critical for the PCCS patients managed at hospitals without ECMO abilities to improve outcomes.

## 1. Introduction

Post-cardiotomy cardiogenic shock (PCCS) is a life-threatening complication, with 0.4 to 3.7% patients reportedly requiring extracorporeal membrane oxygenation (ECMO) [1]. Despite advancements in technology and increased expertise [2], mortality rates among PCCS requiring extracorporeal life support (ECLS) remain high, with survival-to-hospital-discharge rates ranging from 16% to 52%, with fewer than 30% of the centers reporting survival-to-discharge rates above 40% [1]. PCCS remains the most common indication for ECLS in the United States [3], where there are more than 775 hospitals that perform heart surgeries [4]. According to the Extracorporeal Life Support Organization (ELSO) data, 274 centers offer adult ECMO services; however, only 107 ECMO centers have transport abilities. Furthermore, it is not known how many centers in the United States have an ECMO retrieval program. According to The Society of Thoracic Surgeons (STS) data from 2016, more than 70% of coronary artery bypass graft (CABG) surgeries are performed at low-volume centers which often do not have ECMO abilities; therefore, there is a mismatch between the number of patients who may need ECLS post-cardiotomy and the actual number of hospitals which can offer it. The concept of a hub-and-spoke network, as outlined in expert consensus documents, emphasizes the need for well-defined roles, capabilities, and limitations within an institution [2]. In the United States, many hospitals that offer cardiac surgery perform less than 200 operations per year, and it is inevitable that a cohort of patients at these low-volume centers will require mechanical circulatory support [2]. Consequently, it is extremely important that tertiary and quaternary medical centers endeavor to create these hub-and-spoke networks, so that patients undergoing complex surgeries at low-volume institutions can benefit from this potentially lifesaving intervention.

As highlighted in expert consensus documents and guidelines, several key components are essential for the effective use of ECMO in patients with PCCS. These include careful patient selection, the timely application of ECLS, the involvement of experienced ECMO providers, the use of proper precautions and implantation techniques, the application of a well-established weaning protocol, and the ability to recognize when to cease therapy or escalate to more advanced treatments. Together, these elements can enhance the chances of success in this high-risk patient population [2].

Unfavorable outcomes associated with advanced poor end-organ perfusion at the time of ECLS implantation suggest that earlier ECLS implantation is beneficial [5].

According to 2020 EACTS/ELSO/STS/AATS practice guidelines, it is recommended that ECLS support for PCCS is initiated before the onset of end-organ injury or elevated lactate levels (lactate level < 4 mmol/L) in patients who are likely to experience myocardial recovery without uncontrollable bleeding that cannot be surgically repaired. When the likelihood of native myocardial recovery is low, PCCS ECLS is recommended for patients eligible for heart transplant or long-term mechanical circulatory support. The early use of ECLS is also recommended after cardiac surgery in patients with an IABP and optimal medical therapy who fail to wean from CPB or have marginal hemodynamics [2].

ECMO rescue is extremely labor- and resource-intensive [6]. As previously reported, our institution has an ECMO retrieval program in a hub-and-spoke model [7], and, in this analysis, we focused on evaluating the effectiveness and quality of our ECMO retrieval program for post-cardiotomy patients who were cannulated at referral centers and subsequently transported to our hospital, compared to patients who were cannulated at our institution. We specifically assessed the outcomes and incidence rates of adverse events in PCCS patients from these two groups.

## 2. Methods

A retrospective, single-center, observational study was performed on patients who required V-A ECMO support for PCCS from October 2014 to December 2022 at Integris Baptist Medical Center in Oklahoma City. Patients were either cannulated at the referring facilities and were transported to our institution or were cannulated at our hospital. After we obtained approval from the Institutional Review Board of Integris Baptist Medical Center (IRB # 23-003), data were collected via the retrospective review of patient’s electronic medical records.

Patients were cannulated either by one of the physicians from our cardiac intensivist group, which, as previously described, includes physicians with different backgrounds (anesthesia, cardiac anesthesia, medicine, infectious diseases, cardiology, pulmonary, and emergency medicine) [8], or by the referring cardio-thoracic surgeon at the outside institution or from our hospital.

For V-A ECMO cannulations, our practice initially attempted to use a 5 or 7 French (Fr) antegrade reperfusion cannula, followed by a 17 Fr and 23 cm long arterial and 23 or 25 Fr venous femoral drainage cannula, and ECLS support was then initiated. If antegrade cannula placement was unsuccessful, upon return to our institution, we consulted cardiovascular or vascular surgery for antegrade catheter placement via cutdown. In some cases, depending on the vessel size, we used 15 Fr or 19 Fr arterial-sized cannulas. Additional information regarding cannulation strategies can be found in Supplementary Materials (See Supplementary Materials S1).

The decision to place a patient on ECMO at our institution was made by the cardio-thoracic surgeon involved in the patient’s care. Patients from the referral centers were placed on ECMO by the referring cardiothoracic surgeon in the centers that had ECMO cannulation abilities, only after our on-call ECMO intensivist, the referring cardio-thoracic surgeon, and the on-call cardio-thoracic surgeon at our institution had a discussion. Patients who were at centers without ECMO cannulation abilities were placed on ECLS by our ECMO retrieval team. Additional information regarding ECMO retrieval times is available in Supplementary Materials S2.

Excluded from this study were patients below the age of 18 years, patients who required ECMO placement during cardiopulmonary resuscitation (E-CPR), V-A ECMO patients with indications other than PCCS, and individuals that were initially placed on veno-venous (V-V) ECMO.

Data were reviewed for the patients’ demographics, age, body mass index (BMI), V-A ECMO cannulation type (peripheral vs. central), cannulating physician (intensivist vs. surgeon), the location of cannulation, the type of surgery, the urgency of the surgery, other types of mechanical circulatory support (MCS) prior to ECLS, the presence of acute kidney injury (AKI), and the need for continuous renal replacement therapy (CRRT) before ECMO. Lactic acid, platelets, bilirubin, creatinine, and sequential organ failure assessment (SOFA) score prior to cannulation were recorded.

### Statistical Analysis

Data were presented as a mean ± SD, median (IQR—interquartile range), or number (%). The differences between groups were analyzed using the independent *t*-test for continuous variables. The Mann–Whitney U test was used for variables that did not display a normal distribution. Categorical variables were assessed using the chi-square test and Fisher’s exact test when appropriate.

All statistical tests were two-sided, and differences were considered significant when *p* ≤ 0.05. Statistical analyses were performed using the SPSS statistical package (IBM, version 26, New York, NY, USA) and JMP Pro (version 12.0.1, SAS Institute, Cary, NC, USA).

## 3. Results

**Figure 1:**
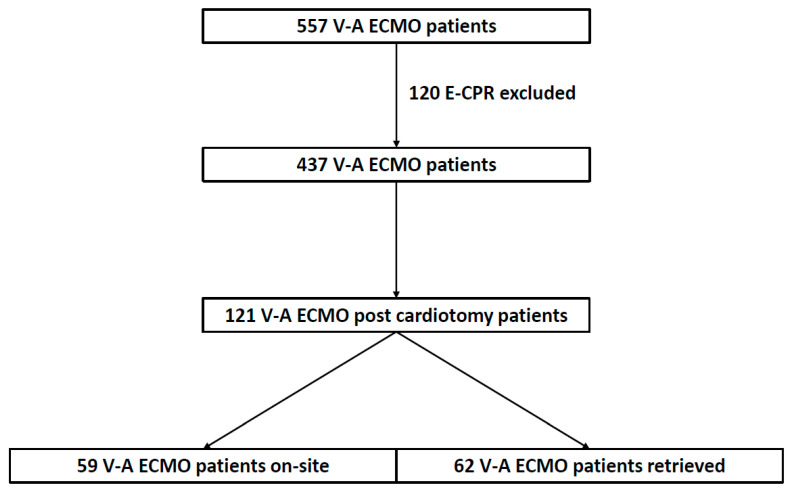
Flowchart diagram of the total number of patients placed on V-A ECMO during the study period, excluding E-CPR cases, focusing on patients who required ECLS for post-cardiotomy cardiogenic shock, with the number of patients retrieved on ECMO vs. cannulated on site. V-A ECMO—veno-arterial extracorporeal membrane oxygenation; E-CPR—ECMO cardio-pulmonary resuscitation.

A total of 557 patients were placed on V-A ECMO during the study period. After excluding E-CPR patients (n = 120), 437 patients were placed on V-A ECMO, of which 121 (28%) patients were post-cardiotomy. Of the 121 patients, 59 (49%) were placed on ECMO at our hospital (on-site group) and 62 (51%) patients were cannulated at the referring institutions and retrieved to our hospital (retrieved group) (Figure 1). Patients were retrieved from our state, as well as neighboring states. Additional information regarding the referring hospitals can be found in Supplementary Materials (See Supplementary Materials S3). Our team traveled to a total of 18 hospitals. The average distance travelled was 70 miles ± 63 (median = 62, IQR = 7.6–107).

The baseline characteristics are presented in Table 1. There were no statistically significant differences in the patients’ characteristics, except a higher level of lactic acid in the retrieved group (8.5 mmol/L ± 5.8) as compared to the on-site group 6.6 mmol/L ± 5) (*p* = 0.04). In the retrieved group, 22% (n = 14) of the patients were transitioned to V-A ECMO straight from cardio-pulmonary bypass (CPB) vs. 27% (n = 16) from the on-site group (*p* = 0.48). There was no significant difference in the time elapsed from the surgical procedure to ECMO implantation between the two groups (retrieved group: median = 1 day, IQR = 0–3 vs. on-site group: median = 1, IQR = 0–2; *p* = 0.50). More patients received intra-aortic balloon pump (IABP) support prior to ECMO in the retrieved group (n = 33, 53%) when compared to the on-site group (n = 21, 36%) (*p* = 0.05). CABG was the most common procedure in both groups [retrieved group: n = 31 (51%) vs. on-site group: n = 28 (47%)], followed by CABG + valve, valve, and others. Of note, 20% or more in each of the groups sustained cardiac arrest prior to ECMO support.

Complications are presented in Table 2. There was no statistically significant different rate of patient-related complications between the two groups. Patients in the retrieved group (n = 15, 24%) had significantly more circuit changes than the on-site group (n = 4, 7%, *p* <0.01).

There was no difference in survival-to-discharge rates between the retrieved group and the on-site group (45% vs. 51%, *p* = 0.53). The overall mortality rate in our study was 52%. Of the patients who expired (n = 63), 50 experienced on-ECMO mortalities (retrieved group: n = 28, 45% vs. on-site group: n = 22, 37%; *p* = 0.38), and 13 had post-weaning mortalities (retrieved group: n = 6, 9.5% vs. on-site group: n = 7, 11%; *p* = 0.7) (Table 3). The ECLS weaning rate was 55% (n = 34) in the retrieved group and 63% (n = 37) in the on-site group (*p* = 0.38). Retrieved patients had a shorter duration of ECMO support (retrieved group: median = 6 days, IQR = 2–13 vs. on-site group: median = 9 days, IQR = 6–15; *p* = 0.05) and a shorter total hospital length of stay (retrieved group: median = 16 days, IQR = 6–27 vs. on-site group: median = 26 days, IQR = 12–39; *p* < 0.01). One patient from the retrieved group was bridged to durable MCS and three were bridged in the other group (*p* = 0.35). None of the patients were bridged to heart transplantation. Patients who experienced shock due to biventricular failure had a 63% survival-to-discharge rate and a 43% survival rate if the etiology of their shock was caused by left ventricular failure, right ventricular failure, or other types (*p* = 0.05).

## 4. Discussion

In this study, we evaluated the outcomes of patients undergoing V-A ECMO support, with a particular focus on the differences between on-site cannulation and retrieval from referring institutions. Our analysis encompassed a total of 121 post-cardiotomy cardiogenic shock (PCCS) patients, with 59 placed on V-A ECMO at our hospital (on-site group) and 62 patients cannulated at the referring institutions and retrieved to our hospital (retrieved group). Our investigation aimed to elucidate any disparities in patient characteristics, clinical outcomes, and complication rates between these two groups. To our knowledge, this is the first study comparing the outcomes of V-A ECMO use in PCCS patients cannulated on site versus patients who were retrieved. In our study, we found no statistically significant difference in survival-to-discharge rates between the groups and no significant difference in the rate of patient-related complications.

As previously outlined in expert consensus documents, the creation of advanced cardiac care systems that offer all aspects of cardiac care, including short-term mechanical circulatory support (ECMO and percutaneous ventricular assist devices), as well as durable ventricular assist devices and transplants, is essential and may affect the outcomes of patients with profound cardiac failure [9].

In a meta-analysis published in 2020 by Kowalewski et al. that included 2235 patients who required post-cardiotomy ECMO, the in-hospital/30-day mortality rate was 66.7%, resulting in a survival-to-discharge rate of 33.3% [10]. Another analysis study conducted by Kakuturu et al., utilizing Nationwide Inpatient Sample data between 2013 and 2018, out of 1,247,835 admissions for cardiac surgical procedures (CABG and/or valve), 0.3% (4475 patients) required ECMO support. The overall survival-to-discharge rate was 57.9% in that cohort, although the study had its limitations in distinguishing between patients who required V-A or V-V ECMO and did not include patients who underwent cardiac surgery at one institution and ECMO at another one (retrieved patients) [11]. In another published report, out of 478 post-cardiotomy patients who required ECLS, 48.3% survived before discharge; however, the authors excluded patients who were placed on ECMO at other institutions, ECLS implanted more than 72 h post-op, and ECMO less than 6 h or patients who underwent thoracoabdominal aortic surgery [12]. In contrast, even though other centers achieved similar survival-to-discharge rates, our cohort included all patients who were placed on ECLS, except those undergoing E-CPR, making it more representative of the population requiring ECMO support.

In our report, the slightly higher incidence of mortality in the retrieved group compared to the on-site group may be explained by the higher incidence of acute kidney injury, as well as higher lactic acid levels in the retrieved group, prior to ECMO cannulation. A systematic review and meta-analysis that included 2877 PCCS patients revealed that an age >65 years, pre-ECMO lactate levels, renal insufficiency, a longer duration of ECMO, and neurologic complications were risk factors associated with increased in-hospital mortality rates [13].

The occurrence of patient-related complications did not show statistically significant differences between the two groups. A total of 18 (15%) neurologic events were encountered, including 9 ischemic strokes in both groups (5 in the retrieved group and 4 in the on-site group), 4 intracranial hemorrhages, and 5 anoxic brain injuries. It is worth noting that 22% (n = 26) of patients, equally distributed between the two groups, had cardiac arrest prior to receiving ECMO support after excluding the patients who were cannulated during active chest compressions (E-CPRs). The rate of neurologic complications in PCCS patients requiring ECLS in a study involving 415 adult patients was 21% and included ischemic stroke (8%), brain death (7.2%), seizures (3.4%), and intracranial hemorrhages (2.7%) [14]. Another study evaluating neurologic complications in 52 patients reported a 38% incidence rate, with ischemic stroke also reported in 25% of the cases [15].

In this analysis, the retrieved group had a slightly higher occurrence of delayed antegrade cannula placement, resulting in an increased incidence of leg ischemia, although the difference was not statistically significant. This can be attributed to the time spent transporting patients back to our institution when antegrade cannulation was unsuccessful at the referring facility. Our practice involves initially attempting antegrade reperfusion cannula placement, followed by arterial and venous femoral cannulation and the initiation of ECLS support. If unsuccessful, upon return to our institution, we consult cardiovascular or vascular surgeons for catheter placement via cutdown.

Interestingly, the retrieved patients required more circuit changes, partially due to the use of a limited number of Cardiohelp machines (Maquet Getinge Cardiopulmonary AG, Rastatt, Germany) by our team for patient retrieval. This necessitated circuit exchanges to accommodate additional patients.

Left ventricular venting was used in 49 (40.5%) patients; however, 41 (34%) patients had pre-ECMO intra-aortic balloon pump or percutaneous left ventricular assist device that was maintained post-ECMO cannulation as a type of LV venting. There is no defined consensus on specific triggers to perform the decompression of the left ventricle in V-A ECMO, but complications of not doing so are well described [16,17]. In our practice, triggers for LV venting include pulse pressure less than 20 mmHg on arterial line (in peripherally cannulated patients), pulmonary wedge pressure greater than 22 mmHg, clinical and radiographic signs of pulmonary edema, the presence of spontaneous echo contrast in the left heart or aortic root, and a lack of aortic valve opening on echocardiogram.

It is important to acknowledge the limitations of our study, including retrospective, single-center, non-randomized design, as well as the limited number of patients.

In conclusion, this study shows that PCCS patients retrieved on ECMO can achieve similar clinical outcomes as those cannulated at experienced medical centers. An established network in a hub-and-spoke model is critical for the PCCS patients managed at smaller hospitals without, or with limited, resources and ECMO abilities in order to improve patient care and outcomes.

## Figures and Tables

**Table 1:** Demographic and pre-ECMO variables of post-cardiotomy cardiogenic shock patients.

Baseline Characteristics	Retrieved Group (n = 62)	On-Site Group (n = 59)	*p*-Value
Age (years)	60.7 ± 12	62.7 ± 11	0.41 ^a^
Gender (male)	46 (74%)	37 (63%)	0.17 ^b^
BMI (kg/m^2^)	31.8 ± 6	31.2 ± 7	0.38 ^a^
V-A ECMO type (peripheral vs. central)	57 (92%) vs. 5 (8%)	55 (93%) vs. 4 (7%)	0.78 ^b^
Proceduralist (intensivist vs. surgeon)	41 (66%) vs. 21 (34%)	38 (65%) vs. 21 (35%)	0.84 ^b^
Cannulation department (ICU vs. OR)	40% vs. 60%	41% vs. 59%	0.08 ^b^
Cardiac arrest pre-ECMO	15 (24%)	12 (20%)	0.61 ^b^
Reoperation	7 (12%)	9 (15%)	0.56 ^b^
Surgical procedure			
CABG	31 (51%)	28 (47%)	0.72 ^b^
Valve	9 (15%)	13 (23%)	0.35 ^b^
CABG + valve	9 (15%)	9 (15%)	1.00 ^b^
Others	12 (19%)	9 (15%)	0.63 ^b^
Urgency			
Emergent	9 (14%)	7 (12%)	0.79 ^b^
Urgent	24 (39%)	29 (49%)	0.28 ^b^
Elective	29 (47%)	23 (39%)	0.46 ^b^
Indication			
Biventricular failure	14 (22%)	16 (27%)	0.67 ^b^
LV failure	26 (42%)	18 (30%)	0.26 ^b^
RV failure	12 (20%)	11 (19%)	1.00 ^b^
Others	10 (16%)	14 (24%)	0.36 ^b^
Open chest	7 (11%)	9 (15%)	0.60 ^b^
IABP pre-ECMO	33 (53%)	21 (36%)	0.05 ^b^
pLVAD pre-ECMO	6 (10%)	4 (7%)	0.56 ^b^
3 or more vasopressors/inotropes	37 (60%)	37 (63%)	0.73 ^b^
Platelets (×10^3^/uL)	128 ± 72	145 ± 76	0.27 ^a^
Bilirubin (mg/dL)	1.7 (1.0–2.5)	1.3 (0.8–2.2)	0.08 ^c^
Creatinine (mg/dL)	1.9 (1.2–2.6)	1.5 (1.1–2.3)	0.33 ^c^
AKI	43 (69%)	33 (56%)	0.09 ^a^
CRRT	6 (10%)	8 (13.5%)	0.50 ^a^
Lactic acid (mmol/L)	8.5 ± 5.8	6.6 ± 5	0.04 ^a^
SOFA score	13.3 ± 3	13.1 ± 3	0.82 ^a^
Peak troponin level (ng/mL)	20.5 (6.7–133.8)	11.2 (2.4–66.1)	0.06 ^c^

ECMO—extracorporeal membrane oxygenation, BMI—body mass index, V-A—veno-arterial, vs.—versus, ICU—intensive care unit, OR—operating room, CABG—coronary artery bypass graft, LV—left ventricle, RV—right ventricle, IABP—intra-aortic balloon pump, pLVAD—percutaneous left ventricular assist device, AKI—acute kidney injury, CRRT—continuous renal replacement therapy, SOFA—sequential organ failure assessment. Definitions are available in Supplementary Materials S4. Data are expressed as means ± SDs, medians (IQR—interquartile range), or numbers (%). *p*-values were generated using the following: ^a^ the independent *t*-test, ^b^ the chi-square test and Fisher’s exact test, and ^c^ the Mann–Whitney U test.

**Table 2:** Complications.

Complications (n = 121)	Retrieved Group (n = 62)	On-Site Group (n = 59)	*p*-Value
Delayed antegrade cannula	10 (16%)	6 (10%)	0.33
Limb ischemia	10 (16%)	5 (8.5%)	0.20
Tamponade	20 (32%)	22 (37%)	0.57
Hemothorax	2 (3%)	4 (6.7%)	0.43
Mediastinal washout	24 (39%)	26 (44%)	0.71
RP bleed	0	2 (3%)	0.23
GI Bleeding	8 (13%)	8 (13.5%)	0.91
Neurologic event	9 (14.5%)	9 (15%)	0.90
LV venting	22 (36%)	27 (46%)	0.25
CRRT	29 (46.8%)	29 (49.1%)	0.79
Bacteremia	2 (3%)	2 (3%)	0.96
Cannula site infection	2 (3%)	1 (2%)	0.59
Circuit change	15 (24%)	4 (7%)	<0.01

RP—retroperitoneal, GI—gastrointestinal, LV—left ventricle, CRRT—continuous renal replacement therapy. Definitions are available in Supplementary Materials S4. Data are expressed as n (%), and *p*-values were generated using the chi-square test and Fisher’s exact test.

**Table 3:** Outcomes.

Outcomes	Retrieved Group (n = 62)	On-Site Group (n = 59)	*p*-Value
Mortality	34 (55%)	29 (49%)	0.53
On ECMO mortality	28 (45%)	22 (37%)	0.38
Post weaning mortality	6 (9.5%)	7 (11%)	0.7
Transition to durable VAD	1 (1.6%)	3 (5%)	0.38

VAD—ventricular assist device, ECMO—extracorporeal membrane oxygenation. Data are expressed as n (%), and *p*-values were generated using the chi-square test and Fisher’s exact test.

## Data Availability

The data that support the findings of this study are available from the corresponding author upon reasonable request.

## Supplementary Materials

The following supporting information can be downloaded www.mdpi.com/10.3390/jcm13113265/s1, The supplementary appendix, containing information such as definitions, details on referring hospitals, additional information regarding ECMO retrieval times, and V-A ECMO cannulation strategies.
