# Supplementary material for: Comparing Outcomes of Post-Cardiotomy Cardiogenic Shock Patients: On-Site Cannulation vs. Retrieval for V-A ECMO Support
Source: J Clin Med. 2024 May 31;13(11):3265. doi: 10.3390/jcm13113265 (PMC11172433; doi:10.3390/jcm13113265)
Supplement: Supplementary file 1 [file jcm-13-03265-s001.zip › jcm-3024157-supplementary.pdf]

Supplementary appendix

S1. V-A ECMO cannulation strategies:

Patients that underwent peripheral V-A ECMO configuration were 112, while 9 patients were cannulated centrally. For peripheral V-A ECMO, the venous drainage cannula sizes used were: 23 French (Fr) venous in 75 patients, 25 Fr in 21, 21 Fr in 14, 18 Fr in 1 and 15 Fr in 1 patient respectively. Arterial return size cannula used were: 17 Fr in 74 patients, 19 Fr in 27, 15 Fr in 7, 23 Fr in 2, 16 Fr in 1, and 20 Fr in 1 respectively. For patients that required central V-A ECMO, the venous cannulas used were 36 Fr in 4, 32 Fr in 1 and 28 Fr in 1. The arterial cannulas used were: 22 Fr in 4 patients, 21 Fr in 1 and 20 Fr in 1 patient. In 3 patients that were cannulated centrally, the cannula size was unknown.

S2. Additional information regarding ECMO retrieval times:

From our experience, as previously published, from the time we accept the patient for retrieval, it takes approximately 45 minutes to one hour until the team leaves the hospital (30 minutes if bed is available immediately). Time to destination depends on the distance travelled and mode of transportation. Once at the outside facility, we evaluate the patient at bedside or in the operating room, discuss with the referring provider and obtain consent from the family. This process usually takes around 30 minutes. V-A ECMO cannulation is performed in approximately 45 minutes to 1.5 hours. This time also includes the initial stabilization post cannulation and securing cannulas for transportation. Once the process is completed, we transfer the patient to the transport stretcher and leave the referring facility once the patient is stabilized enough. From the time we accept the patient for ECMO retrieval to the time we leave the referring facility, it takes a minimum of 3 to 3.5 hours, excluding the time spent to reach the outside institution.

S3. Details on referring hospitals:

- We travelled to 18 hospitals among 3 states, with a median distance of 62 miles (IQR 7.6-107).
- In terms of the referring hospital bed size, based on the classification for Midwest Region from the Agency for Healthcare Research and Quality (US Department of Health and Human Services), there were:
    - 4 urban, non-teaching medium size hospitals (75-174 beds)
    - 11 urban, non-teaching large size hospitals (175+ beds)
    - 1 small urban, teaching hospital (1-249 beds)
    - 2 large, urban, teaching hospitals (375+ beds)
- Overall, out of the referring centers, 8 hospitals had less than 300 beds and 8 were larger than 300 beds, with 4 institutions having 600+ beds. Four out of the 18 centers had ECMO cannulation capability but not long-term ECMO management ability, while the remaining hospitals did not.

S4. Definitions:

- Peak troponin level – highest troponin level during the admission
- Neurological event – includes ischemic stroke, intracranial hemorrhage or anoxic brain injury.
- Gastro-intestinal bleeding – clinical signs of bleeding that started in the GI tract that required blood transfusion.
- Platelets – pre ECMO or within 3 hours post ECMO initiation if not available prior to ECMO cannulation.

- Bilirubin - pre ECMO or within 3 hours post ECMO initiation if not available prior to ECMO cannulation.
- Creatinine - pre ECMO or within 3 hours post ECMO initiation if not available prior to ECMO cannulation.
- Lactic acid - pre ECMO or within 3 hours post ECMO initiation if not available prior to ECMO cannulation.
- SOFA score - pre ECMO or within 3 hours post ECMO initiation if not available prior to ECMO cannulation.
- Type of heart failure - first echocardiogram performed within the first 24 hours from ECMO cannulation, interpreted by a board-certified cardiologist as below:
  - Left ventricular failure – if ejection fraction was below 30%
  - Right ventricular failure – if the report read severely reduced RV function
  - Biventricular failure – a combination of LVEF < 30% and severely reduced RV function
